# Mechanically Reconfigurable Waveguide Filter Based on Glide Symmetry at Millimetre-Wave Bands

**DOI:** 10.3390/s22031001

**Published:** 2022-01-27

**Authors:** Adrian Tamayo-Dominguez, José-Manuel Fernández-González, Oscar Quevedo-Teruel

**Affiliations:** 1 Information Processing and Telecommunications Center, Universidad Politécnica de Madrid, 28040 Madrid, Spain; josemanuel.fernandez.gonzalez@upm.es; 2 Division of Electromagnetic Engineering, KTH Royal Institute of Technology, 10044 Stockholm, Sweden; oscarqt@kth.se

**Keywords:** glide symmetry, filter, mechanical tenability, millimetre-wave

## Abstract

This paper presents the design and fabrication of a mechanically reconfigurable filter at W band based on the concept of glide symmetry. The tunability is achieved by breaking and regenerating the glide symmetry. The filters are made of two glide-symmetric pieces that can be displaced in a certain direction, and therefore, break the symmetry. The high filtering capacity of these designs is demonstrated by simulation and measurement and can also be adjusted mechanically. The transmission level in the manufactured filter varies from a value between −1 and −2 dB when the filter is in the glide symmetry position to values close to −40 dB in the stop-band when it is in the broken symmetry position. The transmission band obtained in the symmetrical mode is around 20%, but, after breaking the symmetry, it is split into two passbands of 6.5% and 11% separated by a stop-band of 6%. The position, bandwidth, filtering level and filter roll-off can be adjusted for both modes of operation by appropriately selecting the unit cell design parameters and the number of unit cells.

## 1. Introduction

Tunning capabilities in microwave and millimetre-wave systems are needed for the new generations of communications. For example, reconfigurable elements in antenna systems allow for modifying the beam pointing direction, changing the shape of the main direction of radiation or changing the shape of the main beam [1]. Reconfigurable filters are also essential for communications systems with dynamic frequency bands or for adjusting the hardware response according to user needs [2,3]. To eliminate the interference in a given band is critical for the proper functioning of a system as well as to reduce the overall terminal costs.

Electrically reconfigurable filters have been developed using, for example, varactors. These devices introduce a variation of the capacity controlled by the voltage, which modifies the resonance frequency of the filters. These filters have been proposed for microwave frequencies from UHF, L and S bands [4,5] to X and K bands [6]. However, finding commercial varactors that operate in millimetre-wave bands is still a challenge. Therefore, when the frequency increases, mechanically reconfigurable devices are typically more cost-effective than electronically reconfigurable ones. For example, reconfigurable filters using screws have been proposed at X and K_u_ bands [7,8]. The depth and thickness of these screws in resonant cavities modify the resonance conditions and produce a frequency shift of the operation band. Similarly, waveguide slot antennas with reconfigurable beams at the K_u_ band have been proposed using screws that modify the phase of the fields in the waveguide [9].

Another alternative that uses mechanical motion to vary device properties is microelectromechanical systems (MEMS). MEMS have mechanical parts that are controlled with electronic circuitry. This type of device is typically based on silicon technology, which allows for very precise manufacturing and high integration. Their application in filters consists of the integration of microactuators that modify the operating band of the filter. MEMS are usually small, so they are a good candidate for filters at high-frequency bands, such as the V band [10,11,12]; however, they have also been used at lower frequency bands, such as X or L bands [13,14]. Unfortunately, for designs not aimed at mass production, MEMS are expensive due to the required complexity and accuracy in the manufacturing.

A promising approach for the design of reconfigurable RF filters is based on the concept of Microwave Photonics (MWP) [15]. This technology integrates optical systems with microwave systems to transfer some of the properties of optical devices to the RF field. In the literature, there are proposals for tunable microwave filters based on this technology [16,17,18,19,20,21]. Despite its potential, this technology still requires further development to improve the characteristics of the devices and currently has some limitations. The level of losses is still high, the reconfiguration capability is limited, and the design, integration and fabrication of these devices is complicated and expensive.

In this field, CMOS-based devices [22,23,24,25,26,27] are also widely employed because of their high level of integration, mass production cost, and combination of RF devices, digital and analogue circuits. However, devices in this technology still consume a lot of power for millimetre-wave band applications.

Another commonly used solution for the design of reconfigurable elements is the displacement of one or more pieces through the structure. In waveguides, this is commonly achieved by moving metal parts or sheets [28,29]. There are proposals in S-band with spring resonator that depending on the pressure applied, the deformation of the springs moves the operating band of the filter [30]. In the terahertz range, there are reconfigurable filters based on polymer Bragg resonators [31]. By controlling the distance between these resonators, the field transmittance response can be modified.

In this paper, we propose a mechanically reconfigurable waveguide filter at the W band based on the concept of glide symmetry. More specifically, we exploit the possibility to open and close a stop-band in a given frequency band by enabling or breaking the symmetry. We demonstrate that, with this technique, the stop-band can be accurately controlled at the W-band. The use of this symmetry for electromagnetic structures was first proposed in the 60 s and 70 s [32,33]. Glide symmetry is a type of higher symmetry that is achieved with a periodic reflection and translation of a given pattern. Recently, the interest in glide symmetric structures has significantly increased due to its interesting properties [34]. Glide symmetry has been proposed to enhance the electromagnetic properties of microwave devices and antennas, for example, to reduce the dispersion in periodic structures [35,36,37]; to significantly increase their equivalent refractive index [38,39,40], anisotropy [41] and magnetic response [42]; and to enhance the attenuation and bandwidth of stop-bands [43,44,45,46,47,48]. With this approach, we deal with the design of millimetre-wave reconfigurable filters in a very simple way. Since it is implemented using a periodic structure, the design difficulty is reduced to properly selecting the design parameters of the unit cell. In addition, the resulting filter can be fabricated in two separate metal parts by traditional CNC machining. Due to the small size of the unit cells in this frequency range, the reconfigurability of the filter is achieved by sliding one of these metal parts over the other by about 1 mm. The control mechanism of the reconfigurable filter is therefore simpler than in proposals where it is necessary to control several components, either electrical (varactors, MEMS, etc.) or mechanical (screws, springs, etc.). Another advantage is the low level of insertion loss since the filter is implemented in full metal, thus improving the performance of other alternatives based on dielectric or semiconductor substrates. In addition, although this paper is focused on the filtering capability, this mechanism also allows to control and reconfigure the phase shift introduced by the structure. This together with the filtering capability is of great interest for the design not only of filters but also of reconfigurable beam antennas.

## 2. Materials and Methods

In this section, we propose and analyse in detail the unit cell structure and the effect of glide symmetry for the filter design. All the simulated results shown in this work are obtained with the commercial electromagnetic tool CST Microwave Studio. All the features of the unit cell have been chosen to make it easily manufacturable with CNC machining in aluminium and to ensure the simplest possible assembly to measure the prototype. For the measurement of the prototype, the Anritsu MS4647B VNA (from 70 kHz to 70 GHz), the Anritsu 3739C mmWave Test Set (from 70 GHz to 110 GHz), two Anritsu 373A mmWave Modules, cables for RF, LO, reference and test signals included in the Anritsu equipment and a WR-10 calibration kit model 27703 from Flann Microwave Ltd. (Bodmin, UK) are used. The measurement setup is shown in Section 3 together with the manufactured prototype. Section 2 includes three subsections detailing the following aspects: Section 2.1 presents the proposed unit cell with generic design parameters. The effect of varying each of these parameters is discussed in depth in this subsection. In Section 2.2, the analysis focuses on two specific configurations of the unit cell: one with double periodicity and one with single periodicity. In both cases, the effect of symmetry breaking is studied. Finally, in Section 2.3, the final unit cell designs modified to best fit the manufacturing and assembly requirements are presented and analysed.

### 2.1. Description of the Unit Cell, Parameter Definition and Generic Analysis

**Figure 1:**
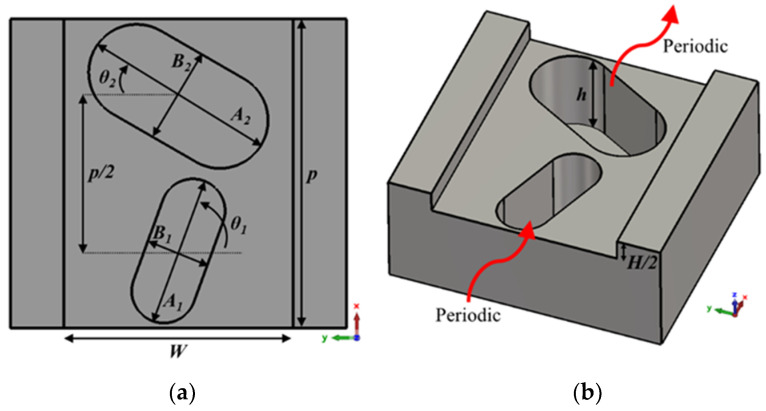
Bottom half of the generic ideal unit cell. (**a**) Top view and (**b**) perspective view. Periodic in *x*-direction.

This section presents the generic unit cell studied in this work. This unit cell consists of a rectangular waveguide in whose top and bottom broad walls are drilled almost elliptical holes whose dimensions and orientation are parameters of study. The holes are stadium-shaped to facilitate their fabrication by CNC machining in aluminium. This shape allows the holes to be fabricated simply with a linear pass of a drill bit of diameter equal to the width of the hole. The lower half of the unit cell is shown in Figure 1, along with the design parameters. Before presenting and analysing how to create the configuration with glide symmetry, in which the upper half of the structure is involved, two design possibilities are described, depending on the parameter configuration chosen. These design parameters are described in Table 1.

**Figure 2:**
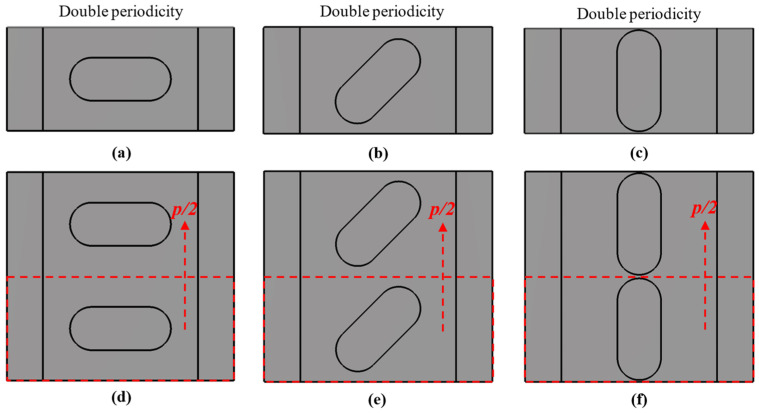
Double periodic configuration for different angles *θ*_1_ = −*θ*_2_ = *θ*. (**a**,**d**) *θ* = 0° (**b**,**e**) *θ* = 45° and (**c**,**f**) *θ* = 90°. (**a**–**c**) Base unit cells of the (**d**–**f**) double periodic unit cells.

**Figure 3:**
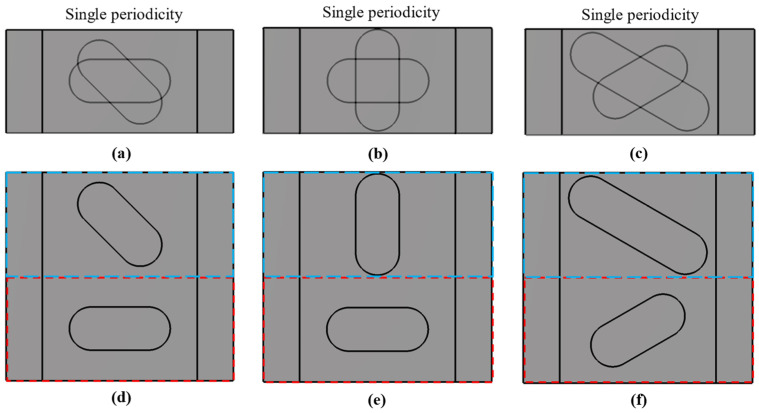
Single periodic configurations for different values of *A*_1_, *A*_2_, *θ*_1_ and *θ*_2_. (**a**–**c**) Overlapping patterns of the hypothetical base unit cells of the (**d**–**f**) single periodic unit cells.

The choice of design parameters can produce two different configurations. If it happens that the parameters *A*_1_ = *A*_2_ = *A*, *B*_1_ = *B*_2_ = *B* and *θ*_1_ = −*θ*_2_ = *θ*, then we have a special case in which this unit cell has double periodicity. This is depicted in Figure 2 for three different cases. It is observed that the three-unit cells in Figure 2d–f are formed by repeating the unit cells in Figure 2a–c. This situation has different properties from the general case, where there is no such double periodicity. This is the case presented in Figure 3, where each half of the unit cell is different and therefore the double periodicity does not appear. This differentiation is fundamental, since for cases with double periodicity, the cell is periodic every *p*/2 step. For the generic case with simple symmetry, the cell is periodic every *p* step.

**Figure 4:**
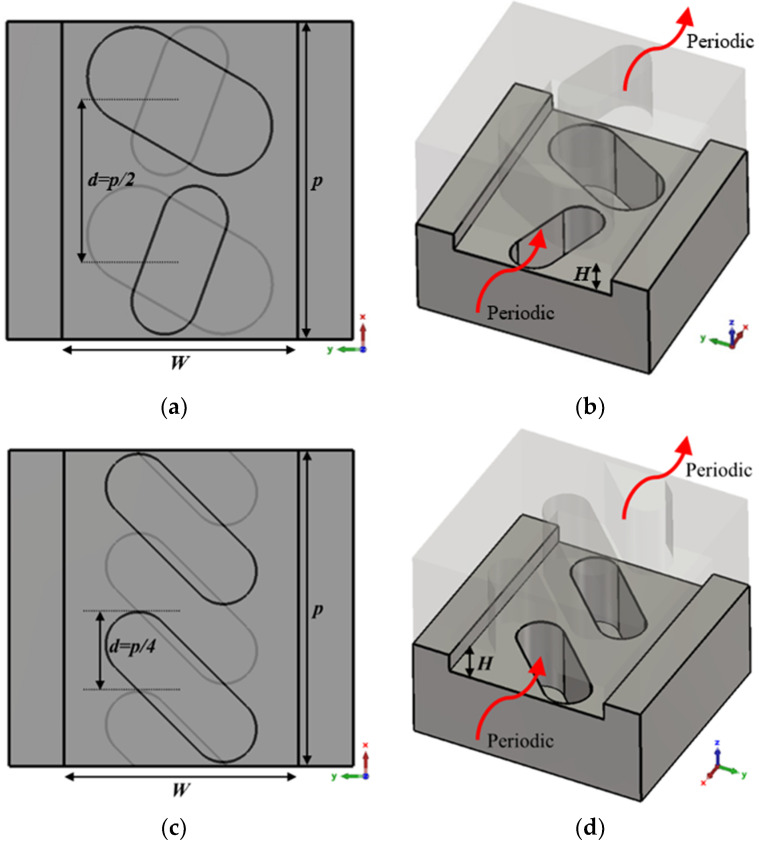
Glide symmetric unit cells for a single periodic configuration (top view (**a**) and perspective view (**b**)) and for a double periodic configuration (top view (**c**) and perspective view (**d**)) periodic in *x*-direction.

To introduce the tuning property, we apply a glide symmetry, so that a vertical mirroring and a half periodicity translation is applied to the described unit cell. Thus, with two pieces facing each other up and down, a displacement *d* is applied to one of the pieces to break or regenerate the symmetry. The glide symmetry condition appears when *d* = *p*/2 for the single periodicity unit cell and *d* = *p*/4 for the double periodicity unit cell. The generation of this glide symmetry is depicted in Figure 4. Figure 4a,b represents the generic case with single periodicity with d = *p*/2, and Figure 4c,d shows the case with double periodicity and d = *p*/4. If *d* differs from these values (or a multiple of them), symmetry breaking occurs, which is maximal when *d* = 0 (or a multiple of *p* or *p*/2, for single or double periodicity respectively). In Section 2.1.1 and Section 2.1.2 we analyse the effect of each of the parameters presented in Table 1.

**Table 1:** Description of the design parameters of the unit cells shown in Figure 1 and Figure 4.

Parameter	Description
*A*_1_	Length of the first stadium-shaped hole.
*A*_2_	Length of the second stadium-shaped hole.
*B*_1_	Width of the first stadium-shaped hole.
*B*_2_	Width of the second stadium-shaped hole.
*θ*_1_	Tilt angle of the first stadium-shaped hole with respect to −*y*-axis.
*θ*_2_	Tilt angle of the second stadium-shaped hole with respect to *y*-axis.
*h*	Depth of stadium-shaped holes.
*p*	Total length of the unit cell. The periodicity for the generic case.
*d*	Displacement along *x*-axis of the upper half with respect to the lower half of the unit cell.
*W*	Width of the rectangular waveguide in which the holes are drilled.
*H*	Height of the rectangular waveguide in which the holes are drilled.

#### 2.1.1. Analysis of Parameter Variation in a Double-Periodic Unit Cell

**Figure 5:**
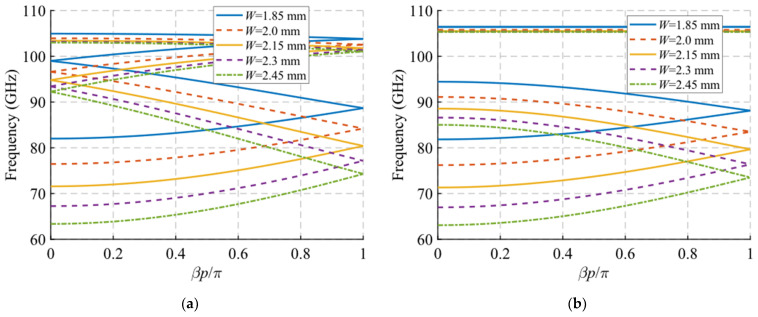
Dispersion diagram of the double-periodic unit cell for various values of *W*. (**a**) Glide-symmetric configuration (*d* = 0.725 mm) and (**b**) maximum rupture of symmetry configuration (*d* = 0 mm).

**Figure 6:**
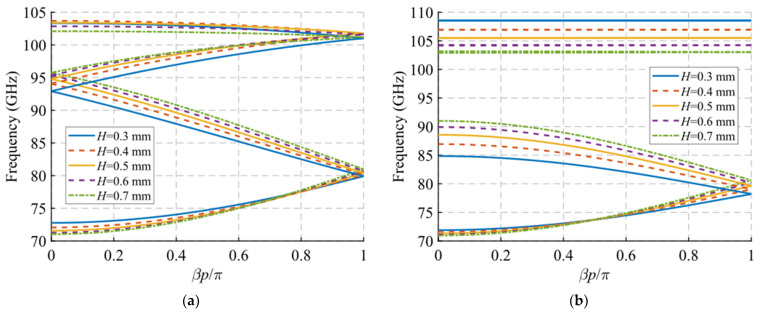
Dispersion diagram of the double-periodic unit cell for various values of *H*. (**a**) Glide-symmetric configuration (*d* = 0.725 mm) and (**b**) maximum rupture of symmetry configuration (*d* = 0 mm).

**Figure 7:**
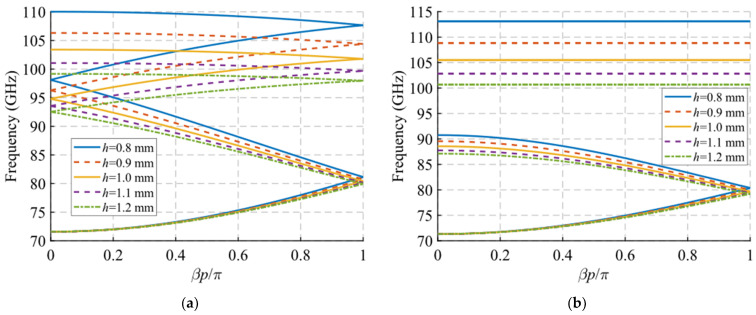
Dispersion diagram of the double-periodic unit cell for various values of *h*. (**a**) Glide-symmetric configuration (*d* = 0.725 mm) and (**b**) maximum rupture of symmetry configuration (*d* = 0 mm).

**Figure 8:**
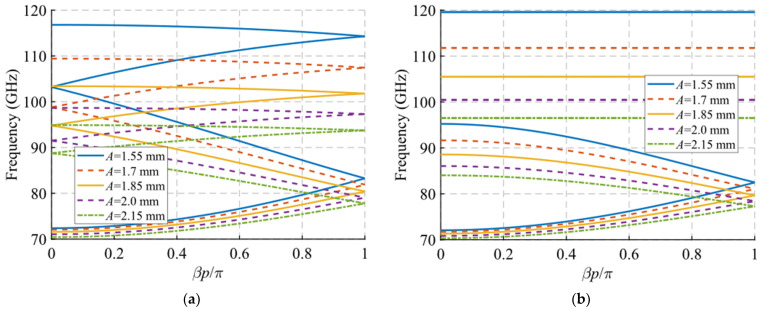
Dispersion diagram of the double-periodic unit cell for various values of *A*. (**a**) Glide-symmetric configuration (*d* = 0.725 mm) and (**b**) maximum rupture of symmetry configuration (*d* = 0 mm).

**Figure 9:**
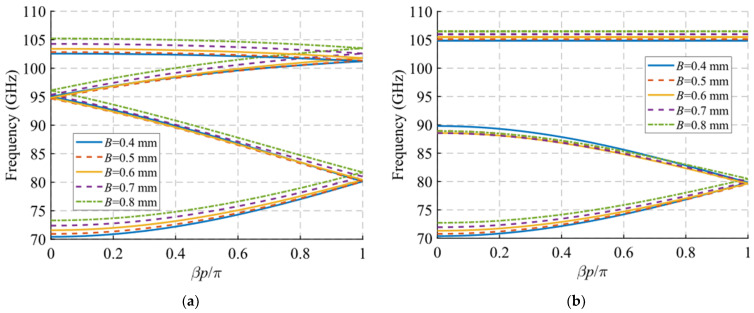
Dispersion diagram of the double-periodic unit cell for various values of *B*. (**a**) Glide-symmetric configuration (*d* = 0.725 mm) and (**b**) maximum rupture of symmetry configuration (*d* = 0 mm).

**Figure 10:**
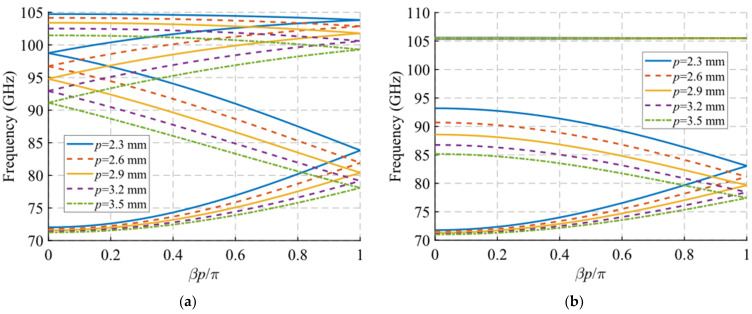
Dispersion diagram of the double-periodic unit cell for various values of *p*. (**a**) Glide-symmetric configuration (*d* = 0.725 mm) and (**b**) maximum rupture of symmetry configuration (*d* = 0 mm).

**Figure 11:**
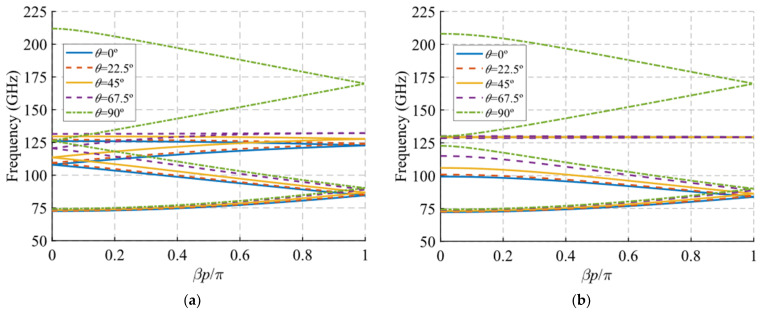

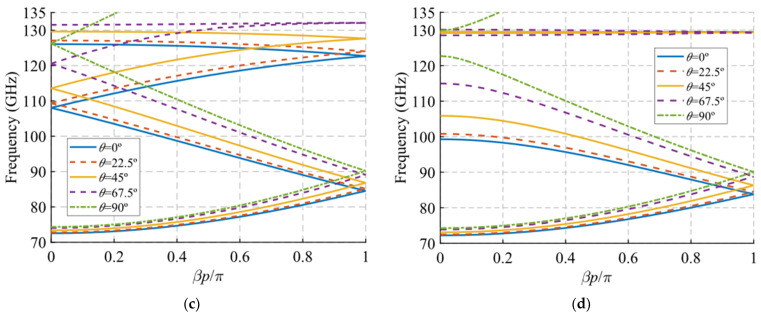
Dispersion diagram of the double-periodic unit cell for various values of *θ*. (**a**) Glide-symmetric configuration (*d* = 0.725 mm) and (**b**) maximum rupture of symmetry configuration (*d* = 0 mm). (**c**,**d**) are zoomed views of (**a**,**b**).

In this section, we analyse the dispersion diagram produced by different parameter configurations for the particular case with double periodicity. Since the periodicity is double, we assume that *A*_1_ = *A*_2_ = *A*, *B*_1_ = *B*_2_ = *B* and *θ*_1_ = −*θ*_2_ = *θ*. For simplicity, we start from a base parameter configuration from which each design parameter is modified one by one. This initial configuration is as follows: *W* = 2.15 mm, *H* = 0.5 mm, *h* = 1 mm, *A* = 1.85 mm, *B* = 0.6 mm, *p* = 2.9 mm and *θ* = 0°. Each configuration is studied for two different values of *d*, corresponding to the glide symmetry arrangement (*d* = *p*/4 = 0.725 mm) and to the maximum symmetry breaking arrangement (*d* = 0 mm). The results obtained by varying *W*, *H*, *h*, *A*, *B*, *P* and *θ* are shown in Figure 5, Figure 6, Figure 7, Figure 8, Figure 9, Figure 10 and Figure 11, respectively. As general characteristics, it is observed that in all cases a four-fold TE_10_ mode is obtained in a glide-symmetric configuration (*d* = 0.725 mm) that splits into two two-fold modes when the symmetry is broken (*d* = 0 mm). In addition, it should be noted that this separation produces the opening of a stop-band in the area of the dispersion diagram *βp*/*π* = 2*n*, where *n* is a natural number (including 0). This point in the diagram corresponds to a phase constant $\beta =\frac{\pi }{p/2}$.

The variation of the waveguide width produces a significant variation of the cut-off frequency of the mode under study, as seen in Figure 5. This effect is to be expected, since the width of a rectangular waveguide is directly related to the cut-off frequency. A variation of *W* from 1.85 mm to 2.45 mm changes the cut-off frequency from 82 GHz to 63 GHz. Therefore, with this parameter, it is possible to control the start frequency of the passband filter. In the case of the waveguide height *H*, it has no significant effect on the glide-symmetric dispersion diagram (Figure 6a), but it does noticeably affect the stop-band aperture (Figure 6b). The lower the waveguide height, the larger the stop-band aperture is observed. A variation from *H* = 0.7 mm to 0.3 mm produces an increase in the stop-band from 12 GHz (12.5%) to 24 GHz (24.5%). The physical phenomenon behind this is that the closer the holes are on the top and bottom faces, the greater their mutual interaction and, consequently, the greater the symmetry breaking effect.

Regarding the depth and size of the holes, a similar pattern is observed in the dispersion diagram when the values of the depth h and the length A of the holes are modified, which are shown in Figure 7 and Figure 8, respectively. In both cases, a downward shift in frequency of the end of the passband is observed when the value of h or A increases. This effect also transfers to the symmetry-breaking configuration (Figure 7b and Figure 8b), where the stop-band also drops in frequency and presents a smaller bandwidth. Therefore, these two parameters can be used to adjust the final frequency of the filter band. The variation of the width B of the holes shown in Figure 9 does not have a significant effect in either of the two situations analysed in this section. However, as will be seen in Section 2.1.2, its effect is fundamental in the unit cell with simple periodicity, since it allows to produce symmetry breaking when the inclination angles *θ*_1_ and *θ*_2_ of the holes are different from each other.

Finally, we consider the variation of the periodicity *p* and the angle of inclination *θ* of the holes. For the parameter *p*, it is observed that its modification does not greatly affect the initial and final cut-off frequencies of the mode in Figure 10a. However, it is seen that the junction points of the forward and backward modes at *βp*/*π* = 2*n* and *βp*/*π* = 2*n* + 1 (corresponding to 0 and 1 in the plot) increase in frequency as the value of p decreases. With a variation of *p* from 3.5 mm to 2.3 mm, the first junction point at *βp*/*π* = 1 moves from 78 GHz to 84 GHz, the first junction point at *βp*/*π* = 0 moves from 91 GHz to 99 GHz and the second junction point at *βp*/*π* = 1 varies from 99 GHz to 104 GHz. This is important for determining the band in which the stop-band appears, since, as shown in Figure 10b, when the symmetry is broken, the stop-band starts to open up at these junction points. In this case, since it has double periodicity, it only opens at the point associated with *βp*/*π* = 2*n*. Therefore, the value of *p* allows the control of both the position and the bandwidth of the stop-band.

Regarding the results for the angle of inclination *θ* shown in Figure 11, something similar occurs. For this case, it was necessary to modify the value of parameter *A* to 1.4 mm to ensure that the holes did not overlap in any case as a function of *θ*. Figure 11c,d shows a zoom of the result in which it is clearly seen that the junction point of the forward and backward modes increase in frequency when the tilt of the holes varies from *θ* = 0° to 90°, i.e., the holes change from horizontal to vertical orientation. When the symmetry is broken, the theta parameter allows the control of the initial frequency of the stop-band. The width of the stop-band increases as the *θ* angle decreases, from 7 GHz (5.8%) for *θ* = 90° until it reaches the maximum width of 30 GHz (26.3%) for *θ* = 0°. This is explained by the fact that the TE_10_ mode propagating inside the structure finds it more difficult to propagate when the notches are placed horizontally, cutting the currents on the broad faces of the waveguides.

Additionally, an interesting phenomenon occurs when the tilt angle *θ* tends to 90°, which is best observed in Figure 11a,b. The TE_10_ modes associated with *θ* values between 0° and 67.5° depicted in the graphic tend to become compressed at frequencies around 130 GHz, where they stop propagating. However, it has been observed that as the value of *θ* approaches 90° this TE_10_ mode merges with a higher mode until they become the same for *θ* = 90°. This is why the TE_10_ mode for *θ* = 90° reaches much higher frequencies. This phenomenon is outside the scope of this study and requires further investigation.

#### 2.1.2. Analysis of Parameter Variation in a Single-Periodic Unit Cell

**Figure 12:**
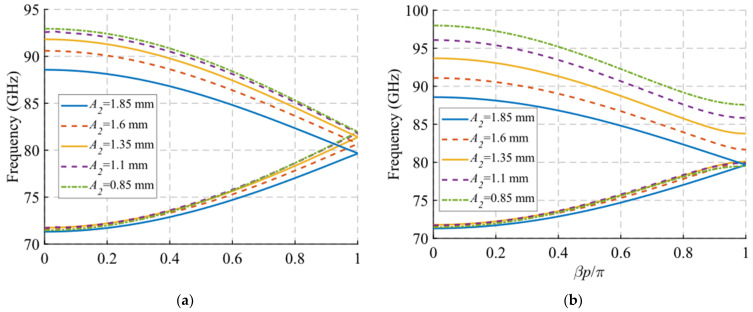
Dispersion diagram of the single-periodic unit cell for various values of *A*_2_. (**a**) Glide-symmetric configuration (*d* = 0.725 mm) and (**b**) maximum rupture of symmetry configuration (*d* = 0 mm).

**Figure 13:**
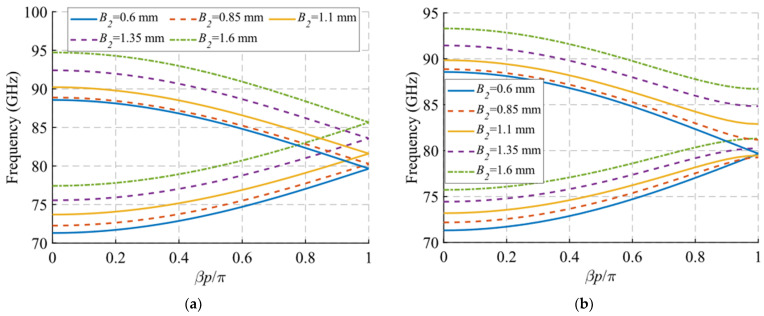
Dispersion diagram of the single-periodic unit cell for various values of *B*_2_. (**a**) Glide-symmetric configuration (*d* = 0.725 mm) and (**b**) maximum rupture of symmetry configuration (*d* = 0 mm).

**Figure 14:**
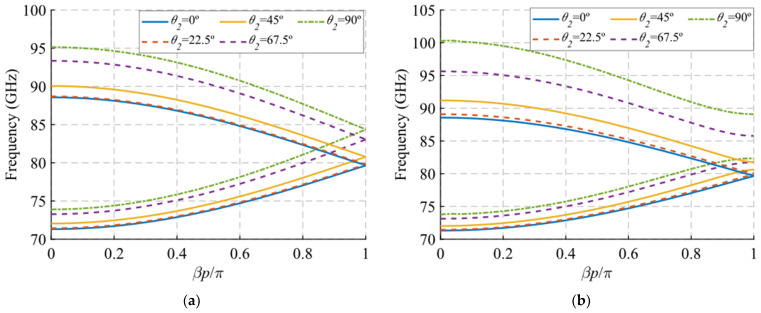
Dispersion diagram of the single-periodic unit cell for various values of *θ*_2_. (**a**) Glide-symmetric configuration (*d* = 0.725 mm) and (**b**) maximum rupture of symmetry configuration (*d* = 0 mm).

In this section, we analyse the dispersion diagram produced by different parameter configurations for the cases with single periodicity. For simplicity, we start from a base parameter configuration from which the parameter *A*_2_, *B*_2_ and *θ*_2_ are modified one by one. We chose only these three parameters because they are the only ones responsible for the single periodicity condition. The initial configuration is as follows: *W* = 2.15 mm, *H* = 0.5 mm, *h* = 1 mm, *A*_1_ = 1.85 mm, *A*_2_ = 1.85 mm, *B*_1_ = 0.6 mm, *B*_2_ = 0.6 mm, *p* = 2.9 mm, *θ*_1_ = 0° and *θ*_2_ = 0°. Each configuration is studied for two different values of *d*, corresponding to the glide symmetry arrangement (*d* = *p*/2 = 1.45 mm) and to the maximum symmetry breaking arrangement (*d* = 0 mm). Since the periodicity is single, we assume that *A*_1_ ≠ *A*_2_ or *B*_1_ ≠ *B*_2_ or *θ*_1_ ≠ −*θ*_2_. The results obtained by varying *A*_2_, *B*_2_ and *θ*_2_ are shown in Figure 12, Figure 13 and Figure 14, respectively. As general characteristics, it is observed that in all cases a two-fold TE_10_ mode is obtained in a glide-symmetric configuration (*d* = 1.45 mm) that splits into two one-fold modes when the symmetry is broken (*d* = 0 mm). In addition, it should be noted that this separation produces the opening of a stop-band in the area of the dispersion diagram *βp*/*π* = 2*n* + 1, where *n* is a natural number (including 0). This point in the diagram corresponds to a phase constant *β* = *π*/*p*.

When the value of *A*_2_ decreases from 1.85 mm to 0.85 mm, the final frequency of the backward mode is shifted upward in frequency. This effect is observed in both the symmetric mode (Figure 12a) and the symmetry-breaking mode (Figure 12b). This shift for the symmetric configuration is from 88.5 GHz to 93 GHz, while with maximum symmetry breaking the variation is larger, from 88.5 GHz to 98 GHz. In addition, as the value of *A*_2_ begins to differ from *A*_1_ = 1.85 mm, the stop-band starts to open. By varying *A*_2_ from 1.85 mm to 0.85 mm, a stop-band with a bandwidth of 8 GHz (9.6%) is achieved. The reason for this is that for values of *A*_2_ equal to *A*_1_, the double periodicity studied in Section 2.1.1 is preserved.

The variation of *B*_2_ produces a frequency shift of the entire mode, not just its final cut-off frequency, as shown in Figure 13. For values of *B*_2_ from 0.6 mm to 1.6 mm, there is an upward frequency shift of 6 GHz for the glide symmetry configuration. This frequency shift is slightly smaller for the configuration with maximum symmetry breaking, where it varies 4.5 GHz. Furthermore, in this symmetry breaking situation, the stop-band opens as the value of *B*_2_ moves away from *B*_1_. In the case of *B*_2_ = 1.6 mm, a stop-band width of 5.4 GHz (6.4%) is obtained. Finally, for the *θ*_2_ parameter in Figure 14, a behaviour similar to that of the *B*_2_ variation is obtained. The full mode shifts upward in frequency as the value of *θ*_2_ increases. In this case, that variation changes slightly throughout the mode. The opening of the stop-band occurs when theta *θ*_2_ differs from the value theta1 *θ*_1_ = 0°, taking a width of 6.7 GHz (7.8%) for *θ*_2_ = 90°.

**Figure 15:**
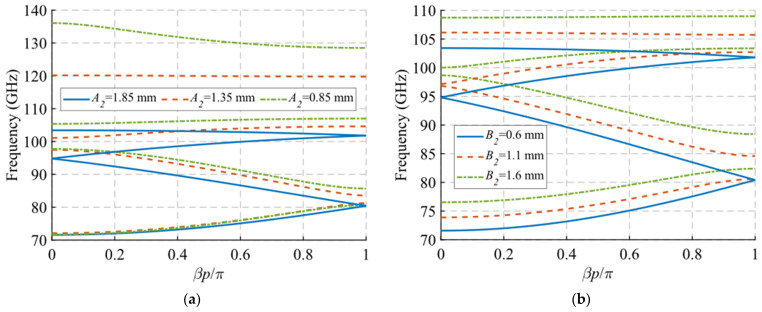

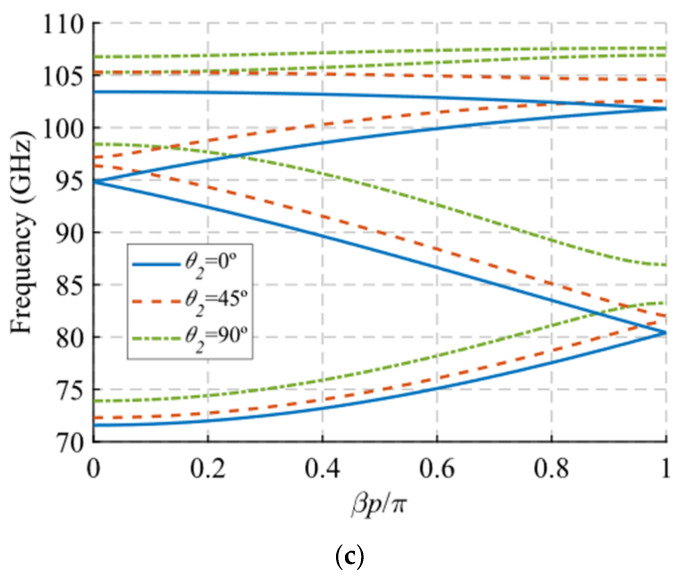
Dispersion diagram of the unit cell for various values of *A*_2_, *B*_2_ and *θ*_2_ when *d* = *p*/4. Transformation from double-periodic to single-unit cells for various values of (**a**) *A*_2_, (**b**) *B*_2_ and (**c**) *θ*_2_.

Before concluding Section 2.1.2, it is necessary to emphasize that a displacement of *d* = *p*/4 as used in the unit cell with double periodicity in Section 2.1.1 is not adequate to achieve a glide-symmetric configuration under the present conditions. The representations in Figure 15a–c illustrate the dispersion diagrams with *d* = *p*/4 = 0.725 mm for different values of *A*_2_, *B*_2_ and *θ*_2_. The values of *A*_1_ = 1.85 mm, *B*_1_ = 0.6 mm and *θ*_1_ = 0° are preserved in all cases. It is clear that, when the values of *A*_2_, *B*_2_ or *θ*_2_ coincide with those of *A*_1_, *B*_1_ or *θ*_1_, the glide symmetry associated with the unit cell with double periodicity is obtained. However, for any other value of these three parameters a total symmetry breaking occurs and the initial four-fold mode separates into four one-fold modes.

### 2.2. Parameter Selection for the Design of Filters

**Figure 16:**
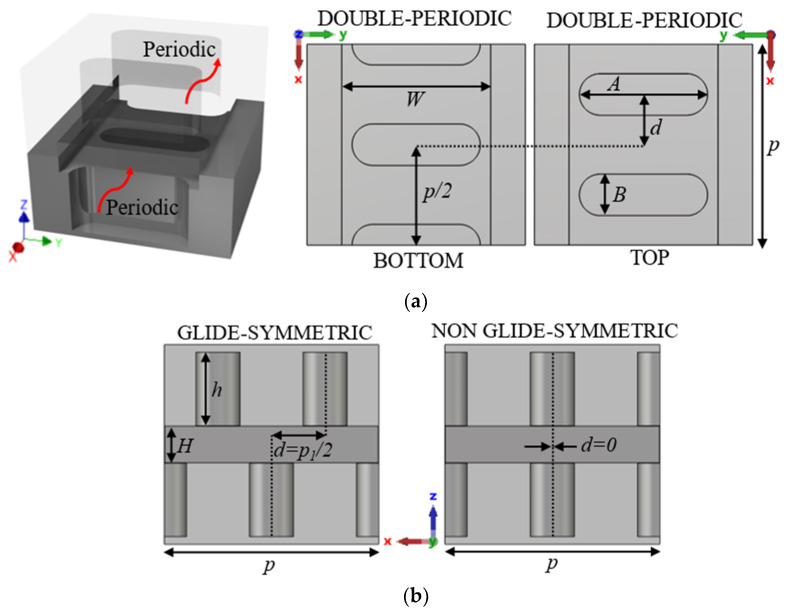
Double periodicity ideal unit cell. (**a**) Perspective, bottom and top views and (**b**) lateral view in glide and non-glide symmetric positions. Periodic in *x*-direction.

**Figure 17:**
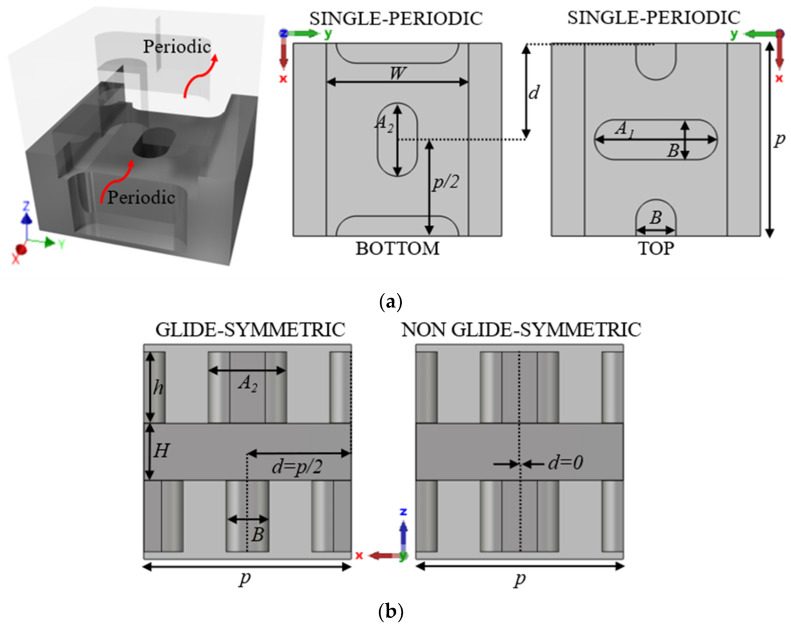
Single periodicity ideal unit cell. (**a**) Perspective, bottom and top views and (**b**) lateral view in glide and non-glide symmetric positions. Periodic in *x*-direction.

In this section, we select two different unit cells for a deeper analysis. The selection takes one unit cell with double-periodicity and another with single-periodicity. For the first case, we choose horizontal (*θ* = *θ*_1_ = *θ*_2_ = 0°) stadium-shaped holes (see Figure 16) and for the second case a horizontal (*θ*_1_ = 0°) and a vertical (*θ*_2_ = 90°) stadium-shaped holes (see Figure 17). The particularity of both designs is that one has double periodicity and the other single periodicity. In glide symmetry conditions, a four-fold TE_10_ mode propagates in the unit cell with double periodicity, but in the single periodic unit cell, a two-fold TE_10_ propagates instead. When one of the pieces is displaced over the other, then the glide symmetry is lost. Therefore, after the displacement, the original four-fold mode is divided into two two-fold modes in the double periodic unit cell and the original two-fold mode in the single periodic unit cell divides into two single-fold modes. This results in the ability to open or close different stop-bands in each design by simply sliding one of the pieces.

Both designs are based on waveguide with a width of 2.15 mm and a length of the unit cell in the periodic direction of 2.9 mm. The values of all design parameters used in the unit cells are shown in Table 2. These values have been carefully chosen to obtain the propagation of the fundamental mode within the W-band, as well as to obtain a wide stop-band generated when the symmetry is broken. Other choices have had their fundamental origin in manufacturing constraints, such as maintaining a margin of at least 0.6 mm between holes, making holes narrow enough to adequately conform to the stadium shape but that can be easily drilled with a 0.6 mm drill bit. The depth of the holes is also adjusted by the manufacturing constraint. The choice of the height of the waveguide lies in obtaining an adequate level of filtration without being too narrow, which can be a problem during the matching of the structure to the transition to WR-10.

#### 2.2.1. Breaking the Symmetry of the Double Periodic Unit Cell

The unit cell with double periodicity is shown in Figure 16. In Figure 16a, it can be seen that it is formed by two pieces, each one of them with double periodicity, which is also positioned in such a way that one piece also forms a glide symmetry with respect to the other when *d* = *p*/4. The separation between pieces corresponds to the height of the waveguide, which in this case is *H* = 0.5 mm. These small heights are necessary to accentuate the effect of the holes. In this ideal design, the gap between the two pieces is sealed on the sides by robust metal, just as in a traditional ideal waveguide.

For the glide-symmetric condition, the displacement of one part over the other is *d* = 0.725 mm, which corresponds to a quarter of the total length *p* of the unit cell. However, since the current structure is equivalent to two consecutive identical unit cells, this displacement is half a unit cell, as required to achieve glide symmetry. The depth of the holes is 1 mm, which ensures that the mode inside them is evanescent, so a small deviation in depth barely affects the behaviour.

**Figure 18:**
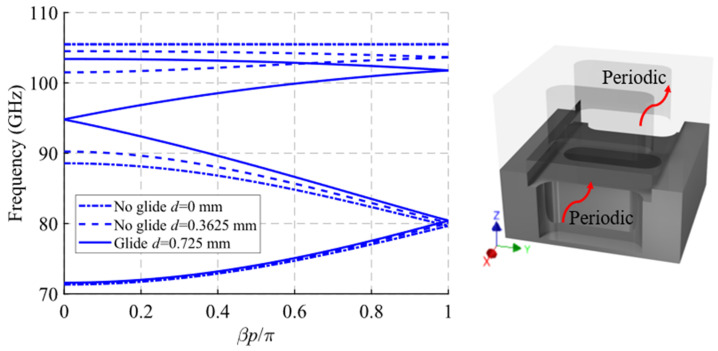
Dispersion diagram of the double periodic ideal unit cell for different stages of break of symmetry. Glide-symmetric for *d* = 0.725 mm and non-glide-symmetric for lower values. *D* = 0 mm for a maximum break of the symmetry.

The dispersion diagram of the unit cell shown in Figure 16 is depicted in Figure 18. It represents three different cases with an offset *d* = 0 mm (maximum symmetry break), *d* = 0.3625 mm (partial symmetry break) and *d* = 0.725 mm (glide-symmetric structure). The double periodic glide symmetry (*d* = 0.725 mm) allows the propagation of a four-fold TE_10_ mode in a band from 71.6 GHz to 103.5 GHz. The break of the symmetry produces a stop-band for *β* = 0 that increases until *d* = 0 mm. At this point, the stop-band covers the maximum band from 88.55 GHz to 105.65 GHz. As the glide symmetry is broken, the original four-fold mode is divided into two two-fold modes.

**Figure 19:**
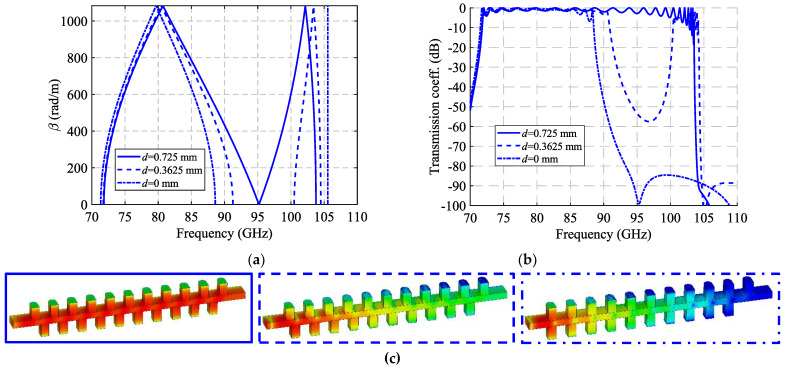
Analysis of the double periodic unit cell for different stages of glide symmetry. (**a**) Phase constant, (**b**) transmission coefficient with *N* = 10 unit cells and (**c**) electric field level inside the structure at 95 GHz.

**Figure 20:**
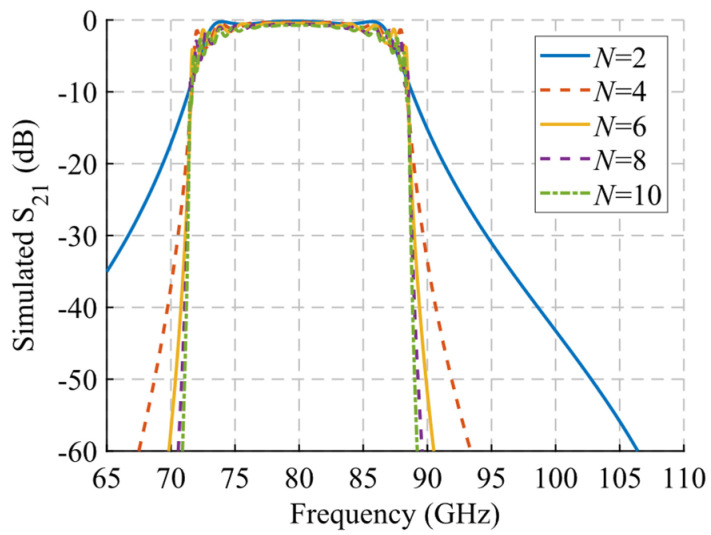
S_21_ parameter for the double periodic ideal unit cell with different repetition number *N* of the unit cell at maximum symmetry breakage (*d* = 0).

A study of the filtering effect produced by symmetry breaking is shown in Figure 19. Figure 19a shows the phase constant, in which the opening of the stop-band is observed as the symmetry is broken. Figure 19b represents the transmission coefficient obtained for each of these values of *d* for a structure with *N* = 10 unit cells, so that an increase of the attenuation in the band corresponding to the stop-band can be seen. Finally, Figure 19c depicts an image of the electric field level inside the structure at 95 GHz. It can be appreciated how the propagation of the electric field is mitigated by modifying the relative position of the stadium-shaped holes. The behavior of the filtering capacity as a function of the number of unit cells used is analyzed in Figure 20. The filter drop becomes more pronounced as more stages are added, as expected. For *N* = 2 a drop of 3.16 dB/GHz is obtained, while for *N* = 10 a value of 33.7 dB/GHz is obtained.

#### 2.2.2. Breaking the Symmetry of the Single Periodic Unit Cell

**Figure 21:**
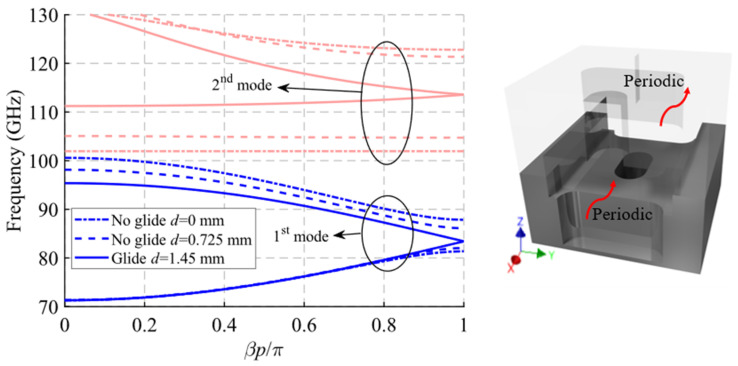
Dispersion diagram of the single periodic ideal unit cell for different stages of break of symmetry. Glide-symmetric for *d* = 1.45 mm and non-glide-symmetric for lower values. *d* = 0 mm for a maximum break of the symmetry.

The unit cell with single periodicity is shown in Figure 17. In Figure 17a, it can be seen that it is formed by two pieces with holes forming a T. Both pieces are positioned in such a way that one piece forms a glide symmetry with respect to the other. The separation between pieces in this case is *H* = 0.8 mm. The lateral sides between the two pieces are also sealed with metal. In this case, the displacement of one part over the other is *d* = 1.45 mm for glide symmetry configuration, which corresponds to half the total length *p* of the unit cell. The dispersion diagram of the unit cell shown in Figure 17 is depicted in Figure 21. It represents three different cases with an offset *d* = 0 mm (maximum symmetry break), *d* = 0.725 mm (partial symmetry break) and *d* = 1.45 mm (glide-symmetric structure). The single periodic unit cell with glide symmetry (*d* = 1.45 mm) allows the propagation of a two-fold TE_10_ mode in a band from 71.2 GHz to 95.4 GHz. The break of the symmetry produces a stop-band for *β* = π/*p* (*p* = 2.9 mm) that increases until *d* = 0 mm. At this point, the stop-band covers the maximum band from 81.3 GHz to 87.9 GHz. The original two-fold mode is divided into two one-fold modes. This is also true for the second mode that propagates in the structure, although this mode is outside the W band.

**Figure 22:**
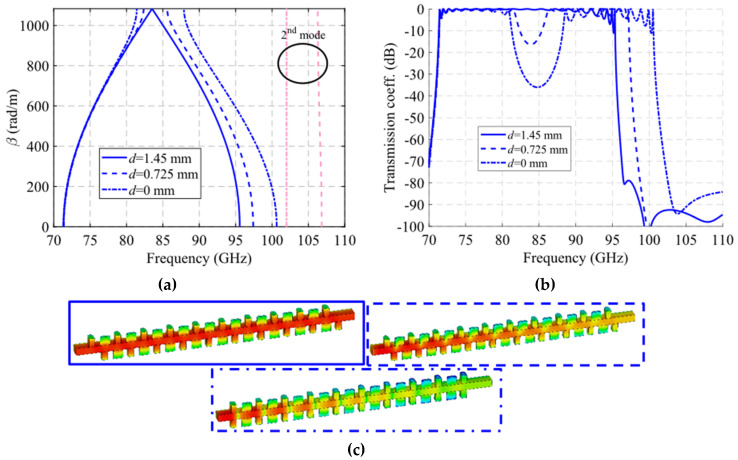
Analysis of the single periodic unit cell for different stages of glide symmetry. (**a**) Phase constant, (**b**) transmission coefficient with *N* = 10 unit cells and (**c**) electric field level inside the structure at 83.5 GHz.

**Figure 23:**
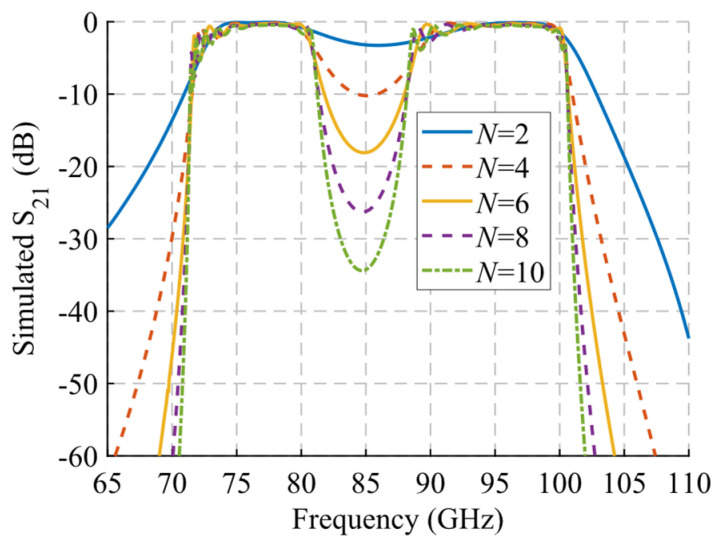
S_21_ parameter for the single periodic ideal unit cell with different repetition number *N* of the unit cell at maximum symmetry breakage (*d* = 0).

A study of the filtering effect produced by symmetry breaking is shown in Figure 22. Figure 22a shows the phase constant, in which the opening of the stop-band is observed as the symmetry is broken. Figure 22b represents the transmission coefficient obtained for each of these values of *d* for a structure with *N* = 10 unit cells so that an increase of the attenuation in the band corresponding to the stop-band can be seen. Finally, Figure 22c depicts an image of the electric field level inside the structure at 83.5 GHz. It can be appreciated how the propagation of the electric field is mitigated by modifying the relative position of the stadium-shaped holes. The behavior of the filtering capacity as a function of the number of unit cells used is analyzed in Figure 23. The amplitude drop becomes more pronounced as more stages are added, as expected. For *N* = 2 a drop of 3.27 dB is obtained at 85.8 GHz, while for *N* = 10 a value of 34.4 dB is obtained.

### 2.3. Final Unit Cells for Manufacturing and Assembly Requirements

**Figure 24:**
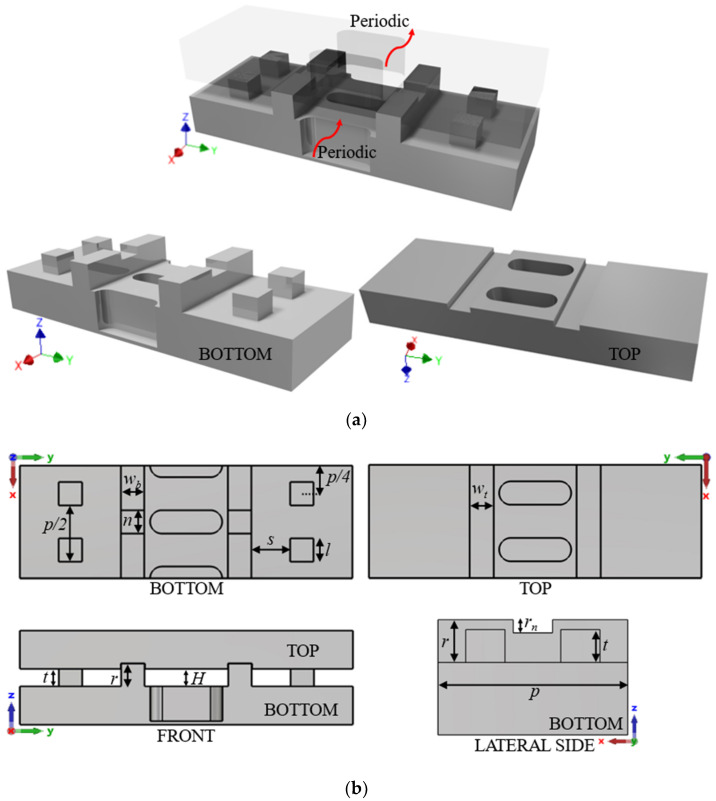
Double periodic glide-symmetric final unit cell. (**a**) Perspective view of the full structure with both top and bottom pieces and (**b**) top, front and lateral views of the complete unit cell.

The final prototypes require an electromagnetic sealing of the interior of the waveguides defined in Figure 16 and Figure 17. For this purpose, new structures have been introduced in the unit cell, as shown in Figure 24. To reproduce the electrical wall on the sides of the waveguide, a metal protrusion 0.6 mm wide has been designed on one face of the waveguide which is inserted into a groove on the other face. The protrusion has a central notch 0.25 mm depth that prevents the propagation of additional modes in the band of interest between the protrusion and the groove. In addition, gap waveguide pins have been introduced to cut off possible field leakage [49,50].

These structures allow the displacement of one piece over the other since there is no crossing of metal parts for any relative position between pieces. However, the modifications slightly break the glide symmetry, as each piece has different shapes. Since most of the electromagnetic fields are confined in the area of the structure that does have glide symmetry, this will not cause issues in the practical implementation. The values of the parameters used in Figure 24 are shown in Table 3.

**Figure 25:**
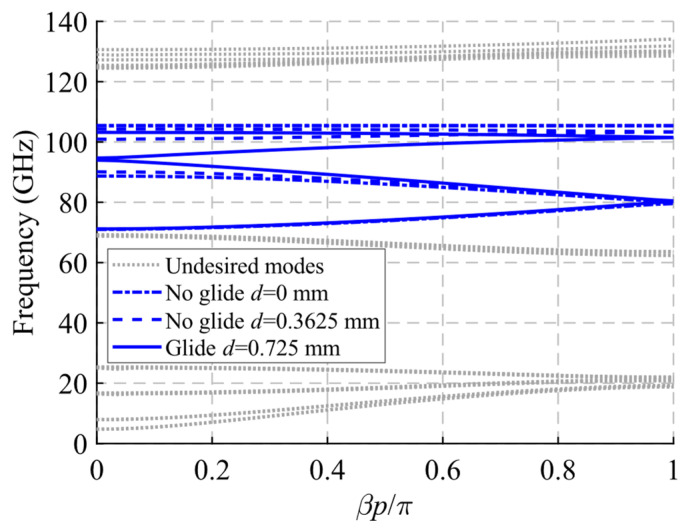
Dispersion diagram of the double periodic final unit cell for different stages of break of symmetry.

**Figure 26:**
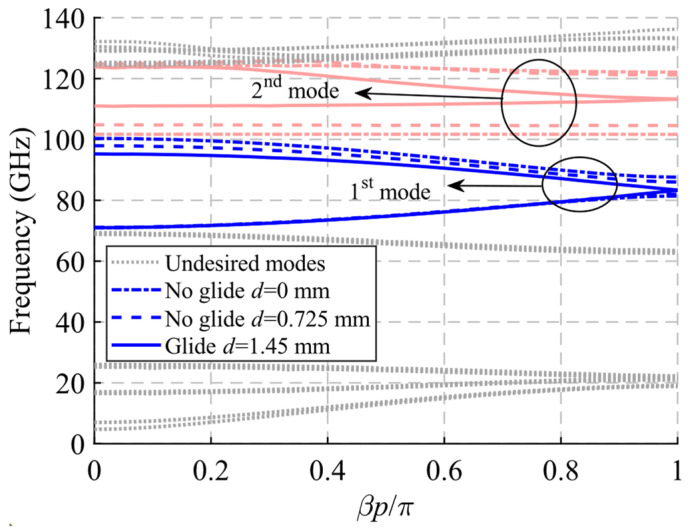
Dispersion diagram of the single periodic final unit cell for different stages of break of symmetry.

The resulting dispersion diagram for the unit cell in Figure 24 is depicted in Figure 25. The modes of the original unit cell remain almost identical. Other unwanted modes appear, but outside the band of interest. The same modifications are applied to the unit cell with single periodicity to ensure the manufacturability of the parts. The behaviour of the dispersion diagram for the modes of interest, 1 and 2, is maintained with respect to the ideal case. Figure 26 shows the modes present in the structure. The unwanted modes are similar to the double periodic unit cell and are outside the band of interest.

**Figure 27:**
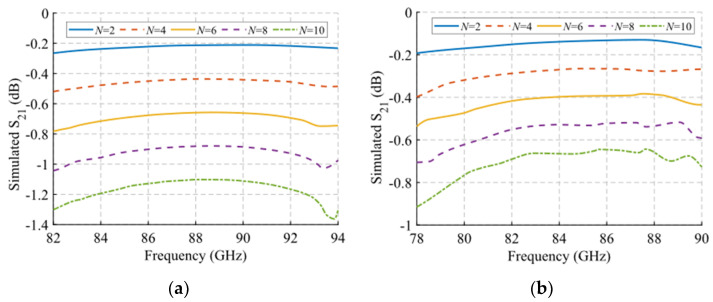
Variation of the S_21_ parameter as a function of the number *N* of unit cells in the filter in glide-symmetric configuration. Filters with (**a**) double-periodic and (**b**) single-periodic unit cells.

An analysis of the losses as a function of the number of unit cells in the filter has been carried out using the final unit cell design. The results are presented in Figure 27. The study has been performed under glide symmetry conditions and considered as an ideal aluminium material with conductivity σ = 3.56 × 10^7^ S/m. A linear increase of about 0.1 dB for each cell added can be observed for the unit cell with double periodicity (Figure 27a). For the unit cell with single periodicity, an increase of about 0.07 dB per unit cell added is obtained (Figure 27b).

## 3. Results

**Figure 28:**
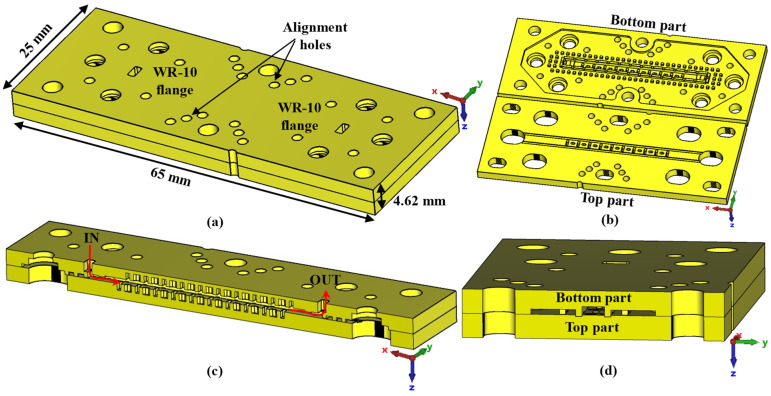
3D model of the final filter designed for manufacturing. (**a**) Complete assembled design, (**b**) interior view of the two pieces that form the filter, (**c**) longitudinal cut of the filter and (**d**) transversal cut.

**Figure 29:**
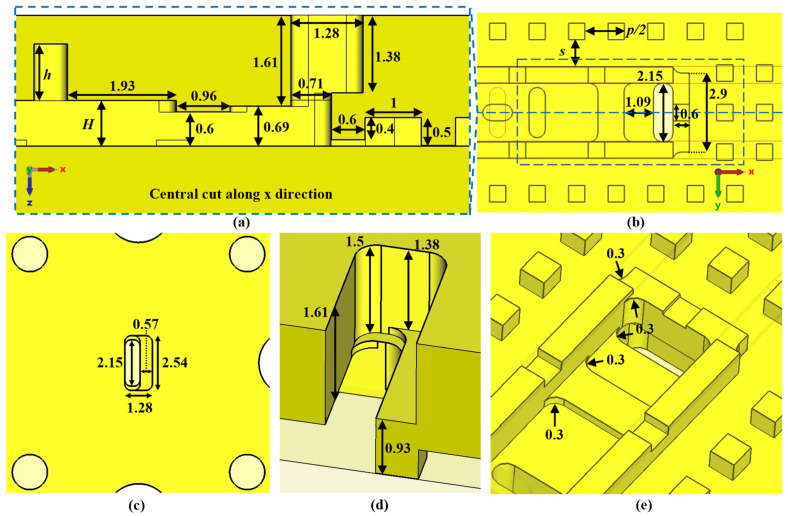
Detailed views of the right-angled bend design to WR-10. (**a**) Lateral cut, (**b**) bottom view, (**c**) top view with the WR-10 input, (**d**) perspective view of the inside of the transition to WR-10 and (**e**) perspective view of the inside.

For this section, only the single periodic prototype has been designed and manufactured, which is shown in Figure 28. The final design includes WR-10 waveguide transitions for connection and measurement. A stepped right-angled bend transition has been designed to access the device perpendicularly. The design details of this bend transition are depicted in Figure 29. This allows the parts to be screwed together in such a way that they can be moved relative to each other. In Figure 28b, the bottom part includes transitions with the holes for the screws and alignment holes for the WR-10 flange. The top piece has only the stadium-shaped holes in the T position and the grooves. These grooves have the additional rail effect to ensure that the parts move in the desired direction. In order to be able to take measurements at specific positions between parts, six additional alignment holes corresponding to six different relative positions have been included in the central part of the design.

**Figure 30:**
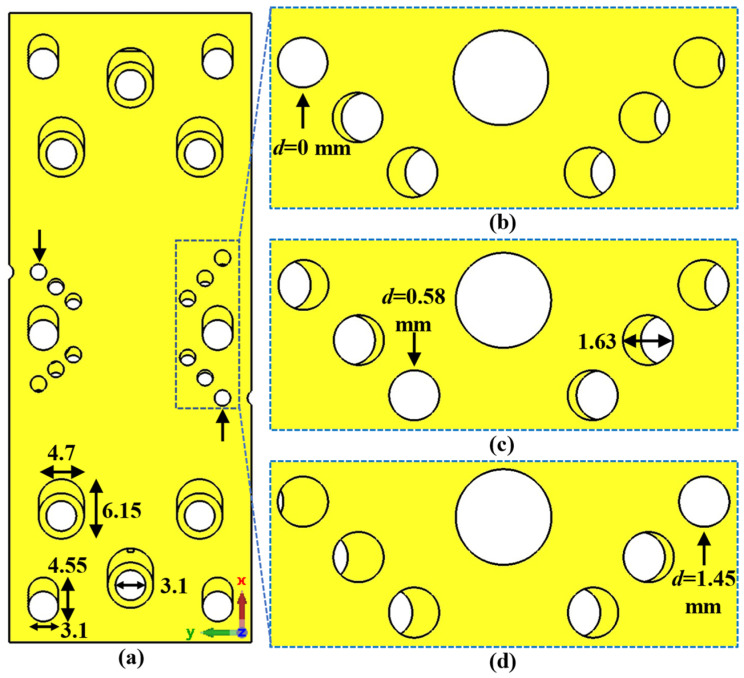
Detailed view of the holes for screws and for the alignment between both pieces that form the filter. (**a**) Top view and (**b**–**d**) detailed view of the alignment holes for different relative positions between pieces.

The alignment holes between parts are located so that, at each relative position associated with *d* = 0 mm up to *d* = 1.45 mm, only one of the six alignment holes coincides exactly with its associated hole in the other part. Therefore, by inserting an alignment pin in these holes we ensure that the relative position between parts is as desired (with a small error of a few tens of microns due to the manufacturing process). Figure 30 shows the dimensions of the holes drilled in the parts for screws and alignment pins and details the operation of the part alignment process. Figure 30b–d show how only one of the alignment holes can be completely traversed by an alignment pin for each position.

**Figure 31:**
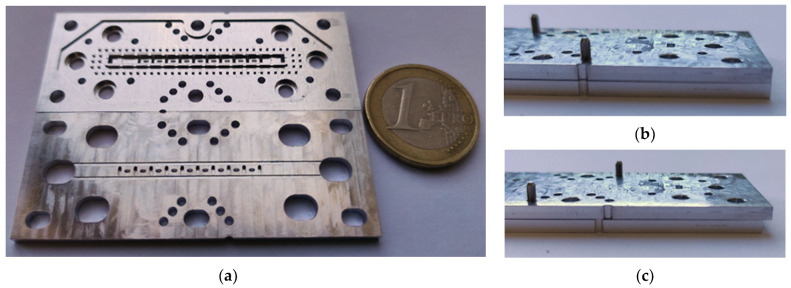
Manufactured single-periodic prototype. (**a**) Inner view of top and bottom pieces, (**b**) pieces in glide-symmetric configuration and (**c**) pieces in broken symmetry configuration.

**Figure 32:**
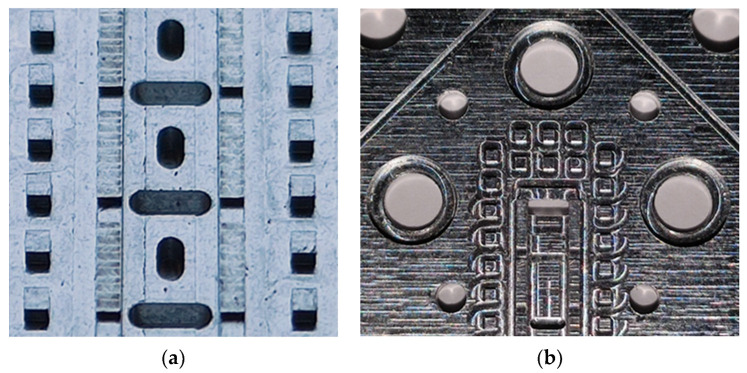
Detailed view of the manufactured single-periodic prototype. (**a**) Zoom of the central waveguide with stadium-shaped holes in T position and (**b**) zoom of the stepped right-angled bend transition to WR-10.

Photographs of the fabricated prototype are shown in Figure 31 and Figure 32. As previously indicated, the prototype has been fabricated on an aluminium part by CNC machining. The process requires high precision so that the interface between the grooves of the upper part and the ridges of the lower part fit perfectly along the entire part, maintaining an error of a few tens of microns. At these frequencies, the problem of high surface roughness can very adversely affect device losses. For this reason, an additional mirror finish has been applied to the most critical surfaces. Figure 31b,c shows the modification of the relative position between parts when pins are inserted in symmetry position or in symmetry breaking position.

**Figure 33:**
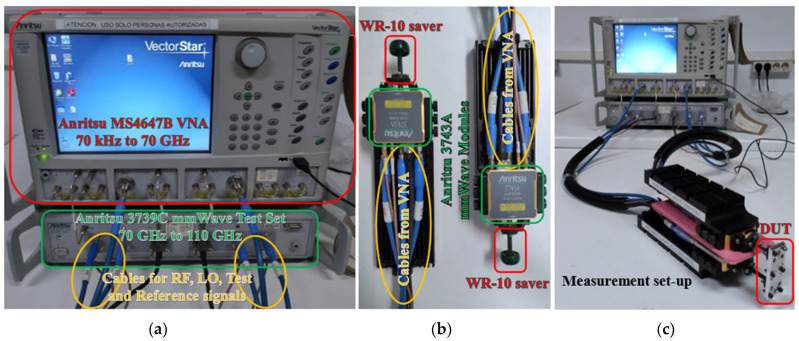
Images of the measuring and assembly equipment. (**a**) VNA and frequency extender, (**b**) measurement heads and (**c**) filter measurement set-up.

**Figure 34:**
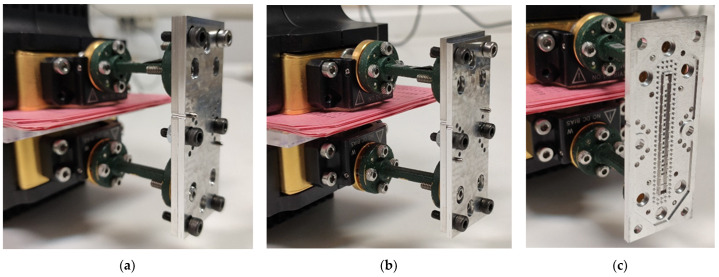
Detailed view of the filter assembly for measurement. (**a**) Filter in glide symmetry position, (**b**) filter in maximum symmetry breaking position and (**c**) view of the inside of the filter connected to the WR-10 flanges.

A detailed view of the stadium-shaped holes in the T position and the transition to WR-10 are shown in Figure 32a,b. Figure 33 shows the equipment used for the measurements and assembly. Figure 34 shows in detail the position of the filter during the measurement process and how the position between parts is controlled to shift from symmetry situation (Figure 34a) to rupture of symmetry (Figure 34b). Figure 34c shows the inside of the filter connected to the WR-10 sections.

**Figure 35:**
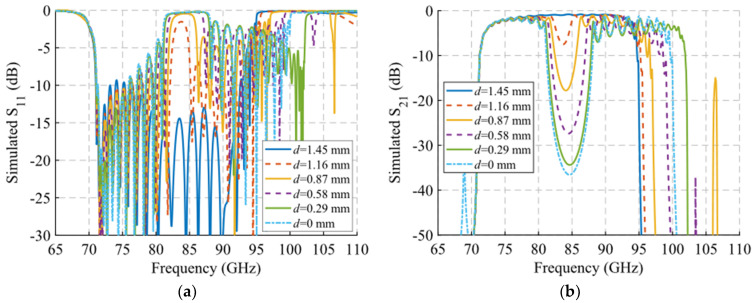

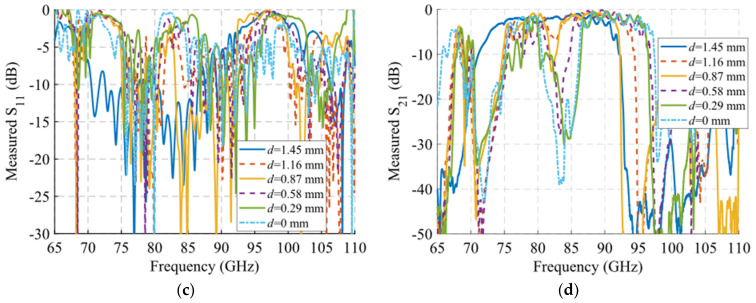
S-parameters of the single-periodic prototype. Simulated S_11_ (**a**) and S_21_ (**b**) results for different displacements *d* values. Measured S_11_ (**c**) and S_21_ (**d**) results for same *d* values.

**Figure 36:**
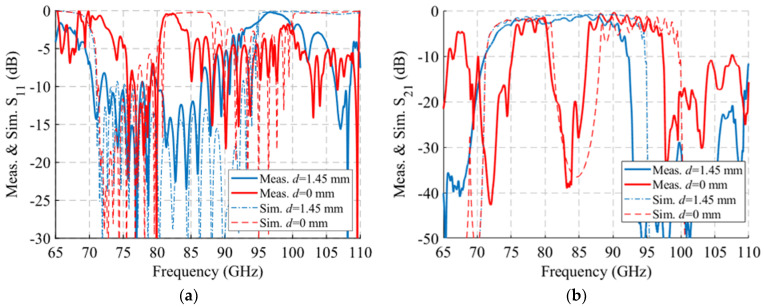
Comparison of the measured and simulated S-parameters of the single-periodic prototype for glide symmetric configuration (*d* = 1.45 mm) and maximum brake of the symmetry (*d* = 0 mm). (**a**) S_11_ and (**b**) S_21_ parameters.

The results of measurements and simulations of this prototype are represented in Figure 35 and Figure 36. The simulated S_11_ and S_21_ parameters are depicted in Figure 35a,b, where it can be seen that for *d* = 1.45 mm there is a band from 73.2 GHz to 92.55 GHz with a reflection coefficient below −10 dB. When the symmetry is broken, for *d* = 0 mm the band splits in two parts with a reflection coefficient below −5 dB from 71.7 GHz to 80.2 GHz and below −2 dB from 89 GHz to 99 GHz. The reflection coefficient increases because the transition to WR-10 is optimized for the glide-symmetric configuration (*d* = 1.45 mm). Regarding the transmission coefficient, taking the values for 84.5 GHz we find that S_21_ = −0.81 dB for *d* = 1.45 mm. The more the parts are displaced, i.e., *d* reduces its value, the more the transmission drops. For a maximum symmetry break (*d* = 0), the value of S_21_ falls to −36.55 dB at 84.5 GHz. This filtering effect occurs between 81 GHz and 88.5 GHz.

**Figure 37:**
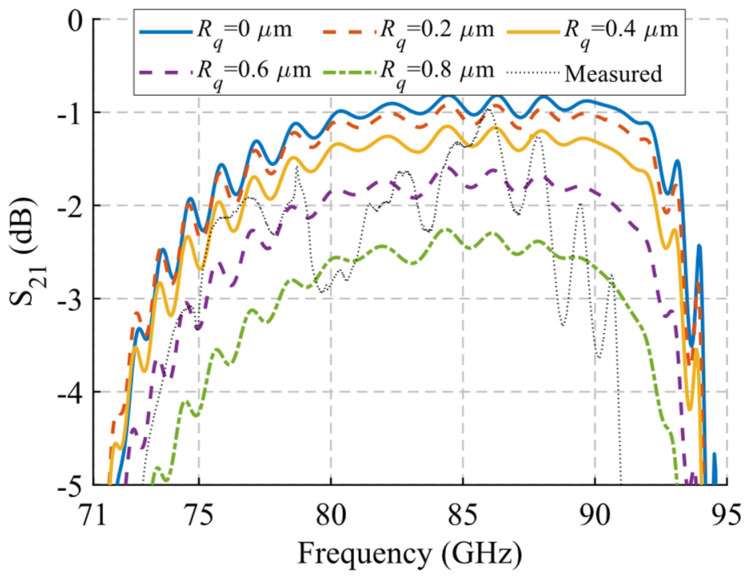
Analysis of the effect of roughness on the transmission parameter of the filter in glide symmetry position. It includes simulations for various roughness RMS *Rq* values overlapping with the measurement.

The measurements for the six positions are shown in Figure 35c,d. When comparing the simulated and measured results, shown overlapped in Figure 36, a great similarity is observed. For the reflection coefficient, depicted in Figure 36a, a level below −10 dB is obtained between 70.5 GHz and 86.5 GHz measured for *d* = 1.45 mm. In simulation, the band covers up to 92.5 GHz, so 27% of the original band has been lost. Despite this, the result is satisfactory since the symmetry-breaking behaviour is perfectly observed when modifying *d* = 0 mm. A noticeable increase in reflection is observed around 82 GHz produced by this symmetry breaking (*d* = 0 mm), accompanied by a modification of the band which coincides to a large extent with the simulation. In transmission (Figure 36b) this is more clearly seen, since in the symmetry situation there is a level above −2 dB between 76 GHz and 89.5 GHz in measurement, but, when the symmetry is broken, the S_21_ level drops to values close to −40 dB around 83.5 GHz. These transmission levels are very similar to those obtained in simulation, although there is a small deviation down in frequency of about 2.4 GHz in both situations. The appearance of spikes and ripples outside the filter band, between 65 GHz and 75 GHz and between 95 GHz and 110 GHz, are due to the propagation within the analysed band of one of the undesired modes shown in Figure 25 and Figure 26. This can be caused by small misalignments between parts due to manufacturing errors. To verify the effect of surface roughness, additional simulations of the device in glide symmetry configuration have been performed by modifying the RMS roughness value *Rq* of the part surfaces. The results are presented in Figure 37, with simulations from *Rq* = 0 to 0.8 µm. It is observed that the measurement is between *Rq* values around 0.4–0.6 µm.

## 4. Discussion

This paper presents a glide-symmetric structure for the design of mechanically reconfigurable filters in rectangular waveguides. Analysis of the unit cells shows the appearance of a stop-band when the upper part slides over the lower part and breaks the glide symmetry. The presence of these symmetries alters the bandwidth properties of the analysed examples and the position and characteristics of the stop-bands that appear when these symmetries are broken. The high filtering capacity of these designs has been demonstrated by both simulation and measurement and can also be adjusted mechanically. Attenuations of around 40 dB or more are observed due to the appearance of the reconfigurable stop-band. The manufactured prototype demonstrates this effect and opens the research of this type of reconfigurable filters using glide symmetry.

## Figures and Tables

**Table 2:** Values of the parameters used in the design of the unit cells shown in Figure 16 and Figure 17.

Parameter	Value (mm)	Parameter	Value (mm)
*A*	1.85	*p*	2.9
*B*	0.6	*A*_1_	1.85
*W*	2.15	*A*_2_	1.1
*H*	0.5 (dp ^1^) and 0.8 (sp ^2^)	*h*	1

^1^ Double periodic structure (Figure 16). ^2^ Single periodic structure (Figure 17).

**Table 3:** Values of the parameters used in the design of the final unit cells with the sealing structure shown in Figure 24.

Parameter	Value (mm)	Parameter	Value (mm)
*r*	*H* + 0.15	*r_n_*	0.25
*t*	0.5	*n*	0.6
*s*	1	*w_b_*	0.6
*l*	0.6	*w_t_*	0.62

## Data Availability

Not applicable.
